# Laparoscopic sacrocolpopexy with mesh fixation: a randomized trial comparing synthetic cyanoacrylate glue to sutures

**DOI:** 10.1007/s00345-025-05885-x

**Published:** 2025-10-07

**Authors:** Gery Lamblin, Graziella Moufawad, Cécile Becque, Chloé Miguet, Stéphanie Moret, Sophie Warembourg, Erdogan Nohuz, Charles-André Philip

**Affiliations:** 1 Department of Gynecological Surgery, Centre de Périnéologie, Hôpital Femme Mère Enfant, Hospices Civils de Lyon, Bron, 69500 France; 2 Université Claude Bernard Lyon 1, Lyon, 69008 France; 3 Department of Obstetrics and Gynecology, Hôpital de la Croix Rousse, Hospices Civils de Lyon, Lyon, 69004 France

**Keywords:** Laparoscopy, Sutures, Pelvic organ prolapse, Tissue adhesives, Surgical mesh

## Abstract

**Purpose:**

To compare laparoscopic sacrocolpopexy using synthetic glue for mesh fixation to laparoscopic suturing in the treatment of pelvic organ prolapse.

**Materials and methods:**

This prospective randomized controlled trial included 54 patients with stage III or IV pelvic organ prolapse who underwent surgical correction and were allocated to two groups: a *glue group* which underwent laparoscopic sacrocolpopexy using synthetic cyanoacrylate glue for mesh fixation and a *sutures group* which underwent the same procedure using suturing for mesh fixation. Operative time, success rate, and perioperative complications were compared between the two groups. Patients were followed up at 1, 12, and 24 months postoperatively.

**Results:**

The median operative time was 108.8 min [83.2–155.6] in the glue group and 111.4 min [90.2–186.2] in the sutures group, without significant difference between the groups. However, the time required for anterior mesh fixation was significantly lower in the glue group compared to the sutures group (4.6 min [0.5–29.6] vs. 25.4 min [1.7–44.7], *p* = 0.0001). The anatomical success rates ranged from 100 to 92.6% at 1 month postoperatively, and from 88.2 to 73.7% at 24 months in the glue and sutures groups, respectively, without statistically significant difference.

**Conclusions:**

The use of synthetic glue in laparoscopic sacrocolpopexy is a safe and effective alternative to suturing. However, larger studies with extended follow-up are required to further assess long-term efficacy and complication rates.

**Supplementary Information:**

The online version contains supplementary material available at 10.1007/s00345-025-05885-x.

## Introduction

Pelvic organ prolapse (POP) is a frequent condition among women, significantly affecting their quality of life, daily functioning, and overall comfort [1]. Laparoscopic sacrocolpopexy is considered the gold standard in the treatment of apical compartment POP [2], resulting in success rates greater than 90%, and providing effective and long-lasting results [3]. Nevertheless, laparoscopic sacrocolpopexy is considered a challenging procedure, requiring advanced surgical skills, deep anatomical knowledge as well as proficiency in suturing and knot tying [1]. Studies suggest a learning curve of at least 15 cases to significantly reduce the operative time [4].

In order to decrease the operative time and the surgical complexity associated with laparoscopic suturing, a synthetic surgical glue can be applied for mesh fixation. The efficacy and feasibility of replacing laparoscopic suturing of certain key points by synthetic glue have been demonstrated in several surgeries [5]. While fibrin-based biologic glues have been historically used, synthetic (cyanoacrelate-based) glue has also been recently implemented. A recent meta-analysis conducted by Tavares et al. on inguinal hernia repair did not find any significant difference between synthetic and biologic-based glue in terms of recurrence, wound infection, hematoma, or seroma formation [6]. Therefore, the authors concluded that glue fixation was safe and effective, and the choice between fibrin- and cyanoacrelate-based glue should depend on cost and availability [6]. Our team has previously conducted a prospective multicenter study to assess the use of cyanoacrylate glue mesh fixation in laparoscopic sacrocolpopexy and found that synthetic glue was safe, time-saving, and effective at 1 year [2]. It facilitated the surgical procedure, decreased operative time, and was associated with good anatomical and functional results [2].

## Objectives

The aim of this randomized controlled trial was to compare operative time, success rate, and outcomes between mesh fixation using synthetic glue and conventional laparoscopic suturing during laparoscopic sacrocolpopexy.

## Materials and methods

### Study design and ethics approval

This prospective randomized controlled trial was conducted between November 2017 and December 2023 at the department of gynecological surgery of the *Hôpital Femme Mère Enfant* in Lyon, France. This study was approved on 08/21/2017 by the *Comité de Protection des Personnes* (CPP) *Nord Ouest III*, in accordance with the ethical standards of the institutional and national research committee, and with the 1964 Declaration of Helsinki and its later amendments. The study was registered in ClinicalTrials.gov (NCT03307824). The primary outcome studied was the operative time in laparoscopic sacrocolpopexy using glue for mesh fixation (Ifabond™), compared to sutures for mesh fixation and prolapse success rates. Secondary outcomes included intra-operative complication rate, early post-operative pain, quality of life, sexual functioning, and urinary incontinence. Follow-up visits were scheduled at 1, 12, and 24 months.

## Participants

Women ≥ 18 years old with apical or anterior POP (POP-Q stage III/IV) who required surgical correction were included. The exclusion criteria comprised POP-Q stages I and II, asymptomatic prolapse, pregnancy, planned childbearing, significant comorbidities (e.g., uncontrolled diabetes, infections, pelvic radiation, pelvic cancer) or POP without functional impairment, diminished leg mobility issues, cognitive impairment, mesh/glue allergies, lack of insurance, or legal guardianship. The eligibility was assessed by the gynecological surgeon. All participants provided written informed consent.

### Randomization and blinding

Patients were randomized to a 1:1 ratio using a computerized system. All surgeries were performed by the same experienced surgeon who had performed more than 30 prior procedures using a standardized laparoscopic approach [7]. In the glue group, laparoscopic sacrocolpopexy was performed using the synthetic glue Ifabond™ and in the sutures group, the procedure was performed using surgical suturing for mesh fixation. Randomization was blinded.

### Surgical procedure

All patients underwent laparoscopic sacrocolpopexy using a standardized technique using polypropylene mesh (Parietex ^™^, Covidien, Mansfield, MA) [8]. Anterior and posterior meshes were fixed with either Ifabond^™^ glue or non-absorbable sutures. Separate anterior and posterior meshes were used when appropriate, no Y-shaped mesh was applied. Patients with stage II rectocele and/or symptoms of dyschezia were candidates for posterior mesh fixation.

For the patients with mesh fixation using glue, a 1.5mL vial was needed for each procedure. The posterior mesh was fixated by applying four drops of 0.2mL of glue on the lower, middle, and upper posterior vaginal wall as laterally as possible [2, 8] and then to the levator ani muscles. The anterior mesh was fixated on the anterior surface of the vagina by instilling glue in a dropwise fashion on six to nine points, with a 0.5 cm minimum distance between two points [8] (see Video in supplementary material).

In all cases, Ifabond™ vaginal mesh adhesive (Peters, France) was used, with the same application technique. In contrast, in the patients randomized to the mesh fixation through suturing, fixation of the posterior mesh was performed via applying three non-absorbable polyethylene terephthalatesutures (Mersuture™) on the same points, and by applying an average of seven nonabsorbable sutures on the anterior vaginal wall.

The mesh was fixed to the sacrum using sutures in both groups. Peritoneum was closed to retroperitonealize the mesh. No tacker or clip were used. The same experienced surgeon with more than 30 procedures who has already reached the learning curve performed all laparoscopic sacrocolpopexies in both groups.

### Physical and chemical properties of the cyanoacrylate glue

Ifabond^™^ is a synthetic sterile, translucent, non-toxic cyanoacrylate tissue adhesive (manufactured by Fimed S.A.S.; CE label, class III) approved for internal use. This low-viscosity glue is sterilized by beta radiation. Upon application to living tissue, it rapidly polymerizes into the flexible adhesive poly n-hexyl-cyanoacrylate [9]. The polymer degrades enzymatically through hydrolysis, producing formaldehyde, hexanol, and cyanoacetic acid. The rate of degradation is inversely proportional to the polymer chain length.

In an animal model, wall reinforcement integrated with tissue more quickly when secured with glue compared to sutures [10]. Tissue formation through cellular colonization occurs around the glue fixation points, allowing the reinforcement to integrate [10]. In a rabbit model, Bellon et al. observed a significantly greater macrophage count between 14 and 90 days following PTFE (polytetrafluoroethylene) reinforcement secured with Ifabond™. Similarly, in a sheep model, the inflammatory response—characterized by increased lymphocyte and plasma cell activity—was significantly greater at two weeks using Ifabond™ compared to staples [11]. Additionally, the apoptotic cell count at 90 days was significantly higher with Ifabond™ than with octyl cyanoacrylate. These responses contributed to significantly stronger fixation at both 14 and 90 days using Ifabond™ [10]. The inflammatory reaction induced by the fixation method, along with the wall reinforcement, supports effective mesh anchorage without causing vaginal ulceration [12].

### Data collection

Pre-, intra-, and post-operative data were collected by the operating surgeon. Patients completed validated questionnaires (PFDI-20, PFIQ-7, PISQ-12, VAS) preoperatively and at 1, 12, and 24 months post-operatively. Anatomical success was defined as POP-Q ≤ 1, and functional success was defined as the absence of bulge symptoms (PFDI-20, Q3). Composite success required anatomical success, no additional surgery, and no bulge symptoms. Mesh shrinkage was defined through IUGA criteria [13]. Demographic and clinical baseline data were collected at enrolment (Table 1). Operative variables included operative time, blood loss, dissection/fixation duration, and complications (Table 2).

**Table 1:** Baseline characteristics of patients

	Glue group sutures *n* = 27	Group *n* = 27	*p*
Age (years)	55.8 [31.2–79.5]	62.0 [28.1–75.1]	0.72
BMI (Kg/m^2^)	24.9 [17.5–34.7]	24.1 [21.0-32.7]	0.51
Parity	2.0 [1.0–4.0]	2.0 [1.0–5.0]	0.65
Number of vaginal deliveries	2.0 [1.0–4.0]	2.0 [1.0–5.0]	0.62
Number of cesarean sections	0.0 [0.0–1.0]	0.0 [0.0–1.0]	0.73
Forceps	10 (40.0)	11 (42.3)	1.00
4th degree perineal tear	9 (34.6)	5 (19.2)	0.35
Menopausal status	16 (59.3)	20 (74.1)	0.39
Urinary incontinence surgery	1 (3.7)	2 (7.4)	1.00
History of hysterectomy	0 (0.0)	0 (0.0)	–
Previous prolapse surgery	0 (0.0)	1 (3.7)	1.00
Stress Urinary incontinence	5 (18.5)	6 (22.2)	1.00
Grade 1	2	0	
Grade 2	2	3	
Grade 3	1	3	
Urinary incontinence by instability	0 (0.0)	3 (11.1)	0.23
Dysuria	18 (66.7)	13 (48.1)	0.27
Urgency urinary incontinence	18 (66.7)	23 (85.2)	0.20
Preoperative UDA^1^	21 (77.8)	23 (85.2)	0.73
closure pressure (cm H2O)	75.0 [36.0-195.0]	59.0 [21.0-110.0]	0.22
maximum flow (ml/sec)	16.8 [7.1–41.0]	22.0 [8.7–47.6]	0.34
Dyschezia	3 (11.1)	2 (7.4)	1.00

Data are median [min-max] or n (%)

^1^ UDA: Urodynamic assessment

**Table 2:** Perioperative outcomes

	Glue group (*n* = 27)	Sutures group (*n* = 27)	*p*
Anterior mesh (frequency)	27 (100)	27 (100)	–
Posterior mesh (frequency)	7 (25.9)	2 (7.4)	0.14
Operative time (min)	108.8 [83.2-155.6]	111.4 [90.2-186.2]	0.74
Rectovaginal dissection duration (min)	12.8 [2.2–36.0]	3.9 [0.6–7.2]	0.22
Levator ani muscle dissection duration (min)	2.2 [1.0-3.7]	4.5 [2.1–6.9]	0.58
Posterior mesh fixation duration (min)	3.5 [0.8–12.4]	7.4 [6.0–8.9]	0.28
Posterior peritonilization duration (min)	3.2 [2.3–7.7]	5.5 [5.2–5.8]	0.28
Duration of posterior glue application (min)	2.4 [1.0-3.5]	–	–
Quantity of posterior glue (mL)	0.5[0.2-1.0]	–	–
Posterior suture fixation duration (min)	–	4.9 [4.9–4.9]	–
Number of posterior sutures (frequency)	–	3.0 [3.0–3.0]	–
Vesico vaginal dissection duration (min)	12.9[3.0-27.5]	8.5 [4.4–27.3]	0.37
Posterior mesh fixation duration (min)	4.6 [0.5–29.6]	25.4 [1.7–44.7]	0.0001
Anterior peritonilization duration (min)	5.5 [0.7–34.7]	4.8 [1.0-50.8]	0.54
Duration of anterior glue application (min)	1.2 [0.1–2.2]	–	–
Quantity of anterior glue (mL)	0.8 [0.2-2.0]	–	–
Anterior sutures fixation duration (min)	–	20.5 [7.8–40.6]	–
Number of anterior sutures (frequency)	–	7.0 [4.0–9.0]	–
Subtotal Hysterectomy	4 (14.8)	1 (3.7)	0.35
Adnexectomy	14 (51.8)	19 (70.4)	0.26
Salpingectomy	4 (14.8)	1 (3.7)	0.35
Transobturator tape	0 (0.0)	0 (0.0)	–
Foley catheter duration (days)	1.0 [1.0–2.0]	1.0 [1.0–2.0]	1.00
Estimated blood loss (mL)	100.0 [0.0-400.0]	100.0 [0.0-300.0]	0.97
D-1 VAS pain scale	2.0 [0.0–5.0]	2.0 [0.0–6.0]	0.93
Hospital stay (days)	2.0 [1.0–3.0]	2.0 [1.0–3.0]	1.00

Data are median [min-max] or n (%)

### Statistical analysis

Statistical analysis was performed using SAS software (SAS Studio 3.8; SAS Institute Inc., Cary, NC, USA). Data did not follow a normal distribution (based on the results of the Shapiro-Wilk test), therefore quantitative results were expressed as median [range] and qualitative results as count and percentage. The variables were compared between both groups using the Mann-Whitney U test for quantitative data and Fisher’s test for qualitative data. The comparison between pre-op and post-op data was performed using the non-parametric Wilcoxon signed-rank test for paired series for quantitative data, and McNemar’s test for qualitative data. Statistical significance was defined as a p-value lower than 0.05.

### Sample size calculation

**Fig. 1:**
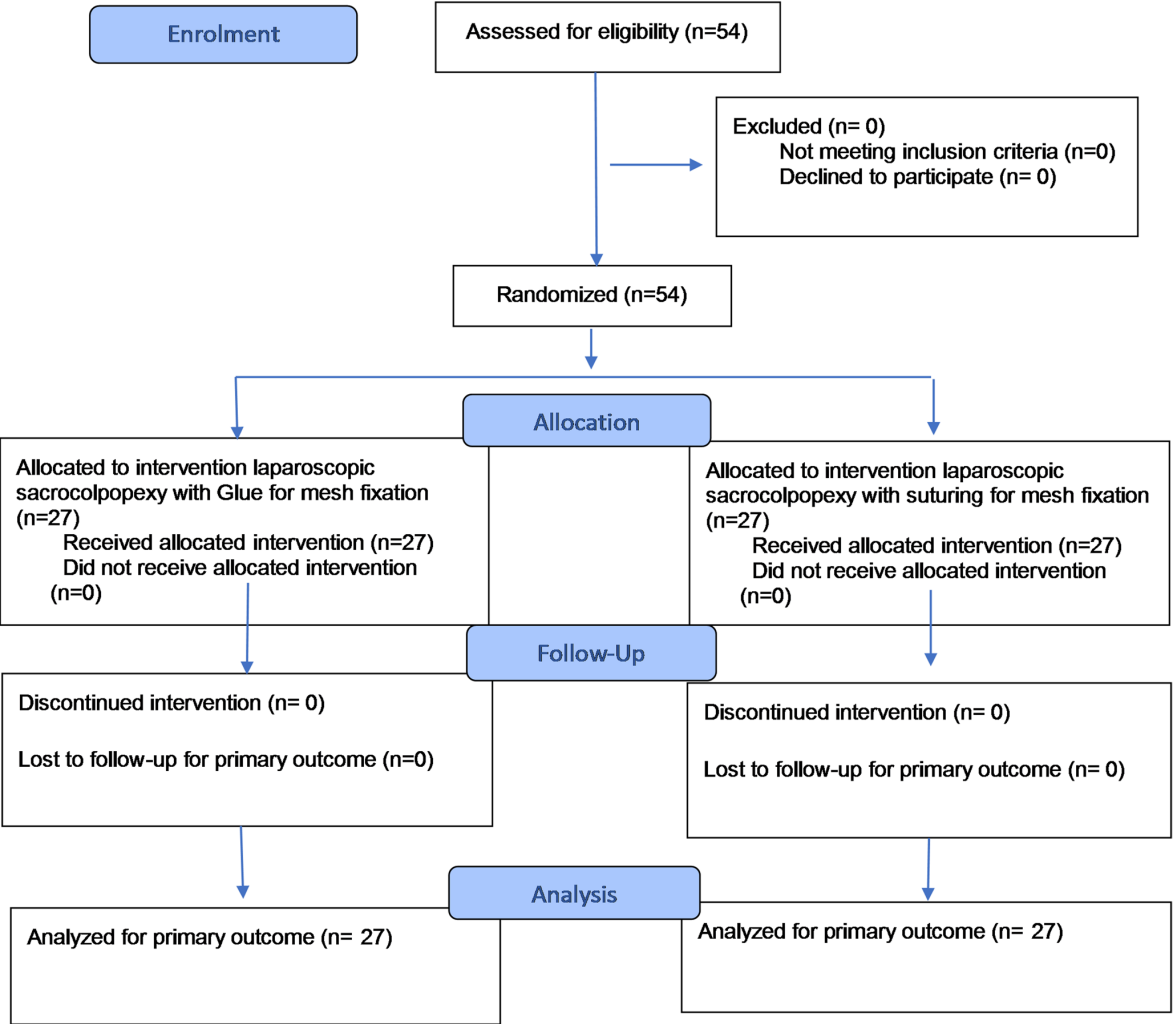
CONSORT 2025 Flow Diagram

It was hypothesized that in the Ifabond™ group, the mean operative time was185 minutes [14]. In our previous study using Ifabond™, the mean operative time was 169 min with reduced operative time with every procedure over the course of the learning curve. Considering the slope of the trend line (a=-0.0248), it can be extrapolated that the operative time was decreased by approximately 10 min over the course of 12 months [2]. Therefore, it was hypothesized that the average operative time in the group treated with Ifabond™ adhesive would be approximately 160 min. Based on this assumption, to achieve an 80% statistical power with an alpha error of 5% and a standard deviation of 30 min, the required sample size is 24 subjects per group, i.e., 48 participants in total. Anticipating approximately 10% dropout prior to the assessment of the primary endpoint, we therefore included a total of 54 patients (as shown in Fig. 1).

## Results

A total of 54 patients were eligible for this study, with 27 patients included in each group. All patients were followed up at 1, 12, and 24 months post-operatively without withdrawal. Baseline characteristics of patients are described in Table 1. The median age of patients was 55.8 years in the glue group and 62 in the sutures group. Previous history of prolapse surgery or incontinence symptoms or surgery are all described in Table 1, as well pre-operative urodynamics.

Intraoperative and early post-operative outcomes are shown in Table 2.

In the glue group and the sutures group, the median operative time was 108.8 min [83.2-155.6] and 111.4 min [90.2-186.2], respectively with no differences between groups. The time required for anterior mesh fixation was significantly shorter in the Glue group compared to the Sutures group (4.6 min [0.5–29.6] vs. 25.4 min [1.7–44.7]; *p* = 0.0001). Both groups had equal amount of estimated blood loss, same average Day 1 pain scale VAS = 2.0, and equal days of hospital stay (Table 2). No bladder injury, rectal injury, hemorrhage, fever, pelvic or urinary infections, or pelvic abscesses were found in any group.

Anatomical success rates (Table 3) for anterior and middle compartments ranged from 100 to 92.6% at 1month and 88.2% − 73.7% at 24 months, in the glue group and the sutures group respectively, without significant difference. At 12 months, cystocele recurrence was higher in the sutures group (56.5*% vs.* 22.7%, *p* = 0.03). No difference was found regarding middle compartment correction. Points Aa and Ba were better corrected in the glue group (-3 vs. -2, *p* = 0.02).

**Table 3:** Anatomical results according to POP-Q classification and stage at 1, 12 and 24 months’ follow-up

	Pre-operatively				1 month			12 months				24 months		
	Glue Group *n* = 27	Sutures Group *n* = 27		p	Glue Group *n* = 27	Sutures Group *n* = 27	p	Glue Group *n* = 22	Sutures Group *n* = 23		p	Glue Group *n* = 17	Sutures Group *n* = 19	
Aa (cm)	3.0 [0.0–4.0]		3.0 [– 2.0-4.0]	0.69	– 3.0 [–3.0 ; –3.0]	– 3.0 [-3.0 ; 0.0]	0.05	– 3.0 [– 3.0 ; 0.0]		– 2.0 [– 3.0 ; 3.0]	0.02	– 3.0 [– 3.0 ; – 1.0]	– 2.0 [– 3.0 ; 3.0]	0.18
Ba (cm)	3.0 [1.0–4.0]		3.0 [– 2.0-4.0]	0.96	3.0 [– 3.0 ; – 3.0]	– 3.0 [-3.0 ; 0.0]	0.05	– 3.0 [– 3.0 ; 0.0]		– 2.0 [– 3.0 ; 3.0]	0.02	– 3.0 [– 3.0 ; 0.0]	– 2.0 [– 3.0 ; 3.0]	0.23
C (cm)	2.0 [-2.0-5.0]		3.0 [– 2.0-6.0]	0.33	– 8.0 [– 9.0 ; – 7.0]	– 8.0 [– 9.0 ; – 7.0]	0.65	– 7.75 [– 9.0 ; – 2.0]		– 7.0 [– 8.5 ; 0.0]	0.33	– 7.5 [– 9.0 ; – 3.0]	– 7.0 [– 9.0 ; – 1.5]	0.12
Ap (cm)	–2.0 [-3.0-1.0]		–2.0 [– 3.0-1.0]	0.41	-3.0 [– 3.0 ; 1.0]	– 3.0 [– 3.0 ; – 2.0]	0.60	– 3.0 [– 3.0 ; 1.0]		– 3.0 [– 3.0 ; 1.0]	0.14	– 3.0 [– 3.0 ; 2.0]	– 2.0 [– 3.0 ; 1.0]	0.06
Bp (cm)	–2.0 [-3.0-1.0]		–2.0 [– 3.0–1.0]	0.41	– 3.0 [– 3.0 ; 1.0]	– 3.0 [– 3.0 ; – 2.0]	0.60	– 3.0 [– 3.0 ; 1.0]		– 3.0 [– 3.0 ; 1.0]	0.14	– 3.0 [– 3.0 ; 2.0]	– 2.0 [– 3.0 ; 1.0]	0.06
D (cm)	-6.0 [-9.0-0.0]		– 6.0 [– 9.0–3.0]	0.93	-9.0 [– 10.0 ; – 7.5]	– 9.0 [– 10.0 ; – 7.0]	0.41	– 8.0 [– 10.0 ; – 7.5]		– 8.5 [– 9.0 ; 1.0]	0.46	-8.5 [– 9.5 ; – 7.5]	– 8.5 [– 10.0 ; – 7.0]	0.80
TVL (cm)	9.0 [6.0–10.0]		9.0 [6.0–10.0]	0.83	9.0 [8.5 ; 10.0]	9.0 [8.0 ; 10.0]	0.17	9.0 [8.0 ; 10.0]		9.0 [6.5 ; 9.5]	0.08	9.0 [8.5 ; 10.0]	9.0 [8.0 ; 10.0]	0.66
Cystocele success rate	–		–	–	27 (100)	25 (92.6)	0.49	21 (95.4)		18 (78.3)	0.19	15 (88.2)	14 (73.7)	0.41
Cystocele	27 (100)		27 (100)	–	0 (0.0)	4 (14.8)	0.11	5 (22.7)		13 (56.5)	0.03	6 (35.3)	10 (52.6)	0.33
stage I	0		1		0	2		4		8		4	5	
stage II	6		6		0	2		1		3		2	4	
stage III	20		18		0	0		0		2		0	1	
stage IV	1		2		0	0		0		0		0	0	
Hysterocele success rate	–		–	–	27 (100)	27 (100)	–	22 (100)		22 (95.6)	1.00	17 (100)	19 (100)	–
Uterine prolapse	27 (100)		27 (100)	–	0 (0.0)	0 (0.0)	–	1 (4.5)		1 (4.3)	1.00	1 (5.9)	2 (10.5)	1.00
stage I	1		1		0	0		1		0		1	2	
stage II	11		8		0	0		0		1		0	0	
stage III	14		16		0	0		0		0		0	0	
stage IV	1		2		0	0		0		0		0	0	
Rectocele success rate	–		–	–	26 (96.3)	27 (100)	1.00	21 (95.4)		21 (91.3)	1.00	15 (88.2)	14 (73.7)	0.41
Rectocele	19 (70.4)		17 (63.0)	0.77	1 (3.7)	2 (7.4)	1.00	4 (18.2)		9 (39.1)	0.19	3 (17.6)	10 (52.6)	0.04
stage I	14		15		0	2		3		7		1	5	
stage II	5		2		1	0		1		2		1	5	
stage III	0		0		0	0		0		0		1	0	

Data are median [min-max] or n (%)

Complications (Table 4) were not significantly different regarding urinary incontinence at 1 and 24 months (15.4% vs. 18.5% at 1 month and 11.8% vs. 21.0% at 24 months). No mesh erosion or exposure were reported in any of the groups. De novo stress incontinence was reported in 2 patients from the glue group and 3 from the sutures group at 1month, 4 in the glue group and 1 in the suture group at 12 months, and none at 24 months.

**Table 4:** Complications at 1, 12 and 24 months’ follow-up

	1 month			12 months				24 months		
	Glue Group *n* = 27	Sutures Group *n* = 27	p	Glue Group *n* = 22	Sutures Group p *n* = 23			Glue Group *n* = 17	Sutures Group p *n* = 19	P
Stress urinary incontinence	4 (15.4)	5 (18.5)	1.00	6 (27.3)		3 (13.0)	0.28	2 (11.8)	4 (21.0)	0.66
Urethral instability incontinence	0 (0.0)	0 (0.0)	–	0 (0.0)		0 (0.0)	–	0 (0.0)	0 (0.0)	–
Dysuria	0 (0.0)	0 (0.0)	–	1 (4.5)		1 (4.3)	1.00	0 (0.0)	0 (0.0)	–
Urgency incontinence	2 (7.7)	5 (18.5)	0.42	6 (27.3)		6 (26.1)	1.00	5 (29.4)	5 (26.3)	1.00
Dyschezia	1 (3.8)	2 (7.4)	1.00	2 (9.1)		1 (4.3)	0.61	0 (0.0)	1 (5.3)	1.00
Vaginal mesh exposure	0 (0.0)	0 (0.0)	–	0 (0.0)		0 (0.0)	–	0 (0.0)	0 (0.0)	–
Mesh shrinkage	0 (0.0)	0 (0.0)	–	0 (0.0)		0 (0.0)	–	0 (0.0)	0 (0.0)	–
Vaginal burning	0 (0.0)	0 (0.0)	–	0 (0.0)		0 (0.0)	–	0 (0.0)	0 (0.0)	–
Urinary infection	0 (0.0)	1 (3.7)	1.00	3 (13.6)		2 (8.7)	0.66	2 (11.8)	1 (5.3)	0.59
Vaginal infection	1 (3.7)	0 (0.0)	1.00	0 (0.0)		1 (4.3)	1.00	1 (5.9)	0 (0.0)	0.47

Data are median [min-max] or n (%)

Functional outcomes are reported in Table 5, and 6. In both groups, symptom improvement and stable PGI-1 scores (median 1.0) were obtained across all time points (Table 6). The SF-12, PFIQ-7, and PFDI-20 scores improved post-operatively without difference between groups. Sexual functioning scores (EVA and PISQ-12) did not change between follow-up and pre-operative assessments in either group.

**Table 5:** Quality of life scores at 1, 12 and 24 months’ follow-up

	Pre-operatively			1 month			12 months			24 months		
	Glue Group *n* = 27	Sutures Group *n* = 27	p	Glue Group *n* = 27	Sutures Group *n* = 27	p	Glue Group *n* = 22	Sutures Group S *n* = 23	p	Glue Group r *n* = 17	Sutures Group *n* = 19	p
PGI-I a	–	–	–	1.0 [1.0 ; 2.0]	1.0 [1.0 ; 2.0]	0.44	1.0 [1.0 ; 6.0]	1.0 [1.0 ; 5.0]	0.97	1.0 [1.0 ; 3.0]	1.0 [1.0 ; 2.0]	0.24
SF-12 b												
Physical score	42.6 [21.6 ; 57.2]	41.4 [23.9 ; 56.6]	0.68	48.1 [32.2 ; 56.8]	43.1 [20.2 ; 52.9]	0.03	45.3 [28.5 ; 57.5]	54.8 [24.4 ; 61.1]	0.08	52.5 [27.4 ; 62.0]	53.1[25.1 ; 58.1]	0.97
Mental score	46.6 [27.2 ; 64.7]	50.9 [23.2 ; 68.9]	1.00	55.1 [24.0 ; 63.8]	55.3 [31.8 ; 72.2]	0.76	56.8 [29.4 ; 67.4]	49.0 [13.6 ; 60.7]	0.03	51.8 [23.8 ; 60.7]	49.8 [33.3 ; 60.7]	0.73
PFIQ-7c	52.4 [0.0 ; 242.8]	66.7 [4.8 ; 236.5]	0.73	9.5 [0.0 ; 90.5]	33.3 [0.0 ; 223.8]	0.25	2.4 [0.0 ; 157.1]	4.8 [0.0 ; 147.6]	0.76	14.3 [0.0 ; 233.3]	11.1 [0.0 ; 161.9]	0.87
UIQ-7	19.0 [0.0 ; 90.5]	16.7 [0.0 ; 100.0]	0.68	0.0 [0.0 ; 66.7]	2.4 [0.0 ; 95.2]	0.35	0.0 [0.0 ; 66.7]	0.0 [0.0 ; 95.2]	0.93	0.0 [0.0 ; 100.0]	4.8 [0.0 ; 100.0]	0.98
CRAIQ-7	4.8 [0.0 ; 76.2]	7.1 [0.0 ; 71.4]	1.00	0.0 [0.0 ; 38.1]	0.0 [0.0 ; 100.0]	0.85	0.0 [0.0 ; 47.6]	0.0 [0.0 ; 85.7]	0.81	0.0 [0.0 ; 100.0]	4.8 [0.0 ; 61.9]	0.83
POPIQ-7	14.3 [0.0 ; 95.2]	38.1 [0.0 ; 100.0]	0.40	0.0 [0.0 ; 52.4]	2.4 [0.0 ; 76.2]	0.56	0.0 [0.0 ; 61.9]	0.0 [0.0 ; 61.9]	0.71	0.0 [0.0 ; 100.0]	0.0 [0.0 ; 19.0]	0.54
PFDI-20d	93.7 [35.4 ; 260.4]	104.2 [23.9 ; 223.9]	0.70	31.8 [0.0 ; 130.2]	67.7 [0.0 ; 148.9]	0.05	39.6 [0.0 ; 167.7]	23.9 [0. ; 193.7]	0.63	37.0 [0.0 ; 166.7]	54.2 [0.0 ; 221.9]	0.46
POPDI-6	50.0[0.0 ; 95.8	50.0 [4.2 ; 100.0]	0.65	0.0 [0.0 ; 62.5]	16.7 [0.0 ; 58.3]	0.04	4.2 [0.0 ; 50.0]	8.3 [0.0 ; 62.5]	0.85	8.3 [0.0 ; 45.8]	8.3 [0.0 ; 70.8]	0.91
CRADI-8	18.7 [0.0 ; 68.7]	18.7 [0.0 ; 78.1]	0.89	9.4 [0.0 ; 43.7]	15.6 [0.0 ; 53.1]	0.32	9.4 [0.0 ; 40.6]	6.2 [0.0 ; 59.4]	0.98	9.4 [0.0 ; 43.7]	12.5 [0.0 ; 71.9]	0.53
UDI-6	33.3 [0.0 ; 95.8]	37.5 [4.2 ; 100.0]	0.95	8.3 [0.0 ; 54.2]	16.7 [0.0 ; 70.8]	0.20	16.7 [0.0 ; 83.3]	8.3 [0.0 ; 100.0]	0.29	12.5 [0.0 ; 91.7]	16.7 [0.0 ; 100.0]	0.39
Sexual relations	14 (53.8)	14 (53.8)	1.00	11 (47.8)	7 (31.8)	0.36	10 (50.0)	10 (52.6)	1.00	10 (47.6)	10 (52.6)	1.00
Sexual relations (scale − 5/5)	–	–	–	0.0 [– 4.8 ; 1.5]	3.5 [0.0 ; 5.0]	0.02	0.0 [– 2.8 ; 3.0]	0.0 [5.0 ; 5.0]	0.52	0.5 [–1.3 ; 5.0]	1.0 [-4.5 ; 5.0]	0.93
PISQ12 e	36.5 [26.0 ; 46.0]	33.0 [18.0 ; 44.0]	0.27	36.0 [30.0 ; 44.0]	43.0 [36.0 ; 48.0]	0.10	38.5 [26.0 ; 42.0]	35.0 [15.0 ; 44.0]	0.71	36.5 [26.0 ; 43.0]	36.0 [18.0 ; 45.0]	0.63
VAS pain scale (0–10)	–	–	–	0.7 [0.0 ; 7.6]	0.5 [0.0 ; 7.9]	0.94	0.0 [0.0 ; 6.0]	0.0 [0.0 ; 8.1]	0.90	0.0 [0.0 ; 8.0]	0.0 [0.0 ; 8.9]	0.95

Data are median [min-max] or n (%)

a PGI-I : Patients Global Impression of Improvement

b SF-12 : Short Form- 12

c PFIQ : Pelvic Floor Impact Questionnaire

d PFDI : Pelvic Floor Distress Inventory

e PISQ : Pelvic Organ Prolapse/Urinary Incontinence Sexual Questionnaire

**Table 6:** Quality of life scores at 1, 12 and 24 months’ follow-up

Glue Group	Preoperative	1 month	p*	12 months	p*	24 months	p*
	*n* = 27	*n* = 27		*n* = 22		*n* = 17	
PGI-I ^a^	–	1.0 [1.0 ; 2.0]	-	1.0 [1.0 ; 6.0]	–	1.0 [1.0 ; 3.0]	-
SF-12 ^b^							
Physical Score	42.6 [21.6 ; 57.2]	48.1 [32.2 ; 56.8]	0.09	45.3 [28.5 ; 57.5]	0.10	52.5 [27.4 ; 62.0]	0.005
Mental Score	46.6 [27.2 ; 64.7]	55.1 [24.0 ; 63.8]	0.03	56.8 [29.4 ; 67.4]	0.003	51.8 [23.8 ; 60.7]	0.11
PFIQ-7^c^	52.4 [0.0 ; 242.8]	9.5 [0.0 ; 90.5]	0.008	2.4 [0.0 ; 157.1]	0.005	14.3 [0.0 ; 233.3]	0.03
UIQ-7	19.0 [0.0 ; 90.5]	0.0 [0.0 ; 66.7]	0.008	0.0 [0.0 ; 66.7]	0.03	0.0 [0.0 ; 100.0]	0.02
CRAIQ-7	4.8 [0.0 ; 76.2]	0.0 [0.0 ; 38.1]	0.17	0.0 [0.0 ; 47.6]	0.08	0.0 [0.0 ; 100.0]	0.80
POPIQ-7	14.3 [0.0 ; 95.2]	0.0 [0.0 ; 52.4]	0.05	0.0 [0.0 ; 61.9]	0.0009	0.0 [0.0 ; 100.0]	0.009
PFDI-20^e^	93.7 [35.4 ; 260.4]	31.8 [0.0 ; 130.2]	< 0.0001	39.6 [0.0 ; 167.7]	< 0.0001	37.0 [0.0 ; 166.7]	< 0.0001
POPDI-6	50.0[0.0 ; 95.8	0.0 [0.0 ; 62.5]	< 0.0001	4.2 [0.0 ; 50.0]	< 0.0001	8.3 [0.0 ; 45.8]	< 0.0001
CRADI-8	18.7 [0.0 ; 68.7]	9.4 [0.0 ; 43.7]	0.08	9.4 [0.0 ; 40.6]	0.38	9.4 [0.0 ; 43.7]	0.10
UDI-6	33.3 [0.0 ; 95.8]	8.3 [0.0 ; 54.2]	0.0004	16.7 [0.0 ; 83.3]	0.01	12.5 [0.0 ; 91.7]	0.006
Sexual relations	14 (53.8)	11 (47.8)	0.56	10 (50.0)	0.32	10 (47.6)	0.56
Sexual relations (scale − 5/5)	–	0.0 [– 4.8 ; 1.5]	–	0.0 [-2.8 ; 3.0]	–	0.5 [– 1.3 ; 5.0]	–
PISQ12 ^c^	36.5 [26.0 ; 46.0]	36.0 [30.0 ; 44.0]	0.26	38.5 [26.0 ; 42.0]	0.15	36.5 [26.0 ; 43.0]	1.00
VAS pain scale (0–10)	–	0.7 [0.0 ; 7.6]	–	0.0 [0.0 ; 6.0]	–	0.0 [0.0 ; 8.0]	–
Satures group	*n* = 27	*n* = 27		*n* = 23		*n* = 19	
PGI-I ^a^	–	1.0 [1.0 ; 2.0]		1.0 [1.0 ; 5.0]		1.0 [1.0 ; 2.0]	
SF-12 ^b^							
Physical Score	41.4 [23.9 ; 56.6]	43.1 [20.2 ; 52.9]	1.00	54.8 [24.4 ; 61.1]	0.002	53.1[25.1 ; 58.1]	0.0008
Mental Score	50.9 [23.2 ; 68.9]	55.3 [31.8 ; 72.2]	0.14	49.0 [13.6 ; 60.7]	0.29	49.8 [33.3 ; 60.7]	0.27
PFIQ-7^c^	66.7 [4.8 ; 236.5]	33.3 [0.0 ; 223.8]	0.05	4.8 [0.0 ; 147.6]	0.05	11.1 [0.0 ; 161.9]	0.01
UIQ-7	16.7 [0.0 ; 100.0]	2.4 [0.0 ; 95.2]	0.05	0.0 [0.0 ; 95.2]	0.04	4.8 [0.0 ; 100.0]	0.02
CRAIQ-7	7.1 [0.0 ; 71.4]	0.0 [0.0 ; 100.0]	0.58	0.0 [0.0 ; 85.7]	0.53	4.8 [0.0 ; 61.9]	0.88
POPIQ-7	38.1 [0.0 ; 100.0]	2.4 [0.0 ; 76.2]	0.01	0.0 [0.0 ; 61.9]	0.003	0.0 [0.0 ; 19.0]	< 0.0001
PFDI-20^d^	104.2 [23.9 ; 223.9]	67.7 [0.0 ; 148.9]	0.0006	23.9 [0. ; 193.7]	0.002	54.2 [0.0 ; 221.9]	0.008
POPDI-6	50.0 [4.2 ; 100.0]	16.7 [0.0 ; 58.3]	< 0.0001	8.3 [0.0 ; 62.5]	< 0.0001	8.3 [0.0 ; 70.8]	< 0.0001
CRADI-8	18.7 [0.0 ; 78.1]	15.6 [0.0 ; 53.1]	0.74	6.2 [0.0 ; 59.4]	0.95	12.5 [0.0 ; 71.9]	0.88
UDI-6	37.5 [4.2 ; 100.0]	16.7 [0.0 ; 70.8]	0.11	8.3 [0.0 ; 100.0]	0.02	16.7 [0.0 ; 100.0]	0.46
Sexual relations	14 (53.8)	7 (31.8)	0.10	10 (52.6)	1.00	10 (52.6)	0.65
Sexual relations (scale − 5/5)	–	3.5 [0.0 ; 5.0]	-	0.0 [– 5.0 ; 5.0]	–	1.0 [-4.5 ; 5.0]	–
PISQ12 ^e^	33.0 [18.0 ; 44.0]	43.0 [36.0 ; 48.0]	0.06	35.0 [15.0 ; 44.0]	0.95	36.0 [18.0 ; 45.0]	0.67
VAS pain scale (0–10)	–	0.5 [0.0 ; 7.9]	–	0.0 [0.0 ; 8.1]	–	0.0 [0.0 ; 8.9]	–

Data are mean (± standard deviation) or n (%)

^a^ PGI-I : Patients Global Impression of Improvement

^b^ SF-12 : Short Form- 12

^c^ PFIQ : Pelvic Floor Impact Questionnaire

^d^ PFDI : Pelvic Floor Distress Inventory

^e^ PISQ : Pelvic Organ Prolapse/Urinary Incontinence Sexual Questionnaire

*p : compared to preoperative data

## Discussion

Laparoscopic sacrocolpopexy is the gold standard in the surgical management of pelvic organ prolapse [15]. It is an effective procedure with long lasting results and can be of great interest in the management of POP in sexually active women [15]. However, from a surgeon’s point of view, this procedure requires expertise and excellent surgical skills especially regarding tissue dissection and mesh fixation via suturing [16]. Studies have shown that at least 18–40 cases of laparoscopic sacrocolpopexy are required for a surgeon to become proficient [16]. Usually, sutures are the main technique for mesh fixation; however, several other techniques have been proposed including tackers, or synthetic glue [17, 18]. While no advantage of tacks has been shown as compared to mesh fixation via suturing [17], glue mesh fixation has been reported to be safe and feasible, in comparison to tacker fixation [18]. Since mesh fixation constitutes a major operative time during the procedure, using synthetic adhesive for mesh fixation instead of sutures significantly decreases the operative time [15].

This randomized study aimed to compare the operative time of mesh fixation during laparoscopic sacrocolpopexy using glue to using sutures for mesh fixation. The anatomic success rate, functional results and complications were also compared.

The study included 54 patients, equally and randomly divided between both groups. Only one experienced surgeon who has already obtain the learning curve (more than 100 procedures), operated on all the patients, in one single center, with the same peri-operative conditions, contributing to decreasing the difference and the variables that might affect the results.

Both groups had approximately an equal operative time, without any statistical significance (109 min for the glue group vs. 111 min for the sutures group). An important advantage in the glue group was the significantly decreased time required for the anterior mesh fixation (4.6 min vs. 25.4 min) with a p-value = 0.0001. However, given the significant decrease in anterior mesh fixation duration, glue mesh fixation can be an effective alternative to enhance surgical workflow. Glue mesh fixation reduces operative time [15], although Willecoq et al. concluded in their study that using glue for mesh fixation did not decrease overall operative time but decreased operative time in a small sample of patients and in the beginning of the learning curve [19]. In our case, the gynecological surgeon was already experienced and has already overcome his learning curve. Glue mesh fixation can therefore be of great importance for young surgeons, who do not yet have sufficient expertise.

The efficacy of synthetic glue mesh fixation was similar efficacy regarding complications and outcomes; equal amount of blood loss was found as well as similar post-operative pain levels (VAS = 2.0 on day 1 in both groups), and equal hospital length stay (2 days) without additional perioperative morbidity.

Regarding anatomical success, both groups exhibited favorable outcomes for the anterior and middle compartments with a success rate ranging from 100 to 92.6% at 1 month post-operatively to 88.2% and 73.7% at 24 months, in the glue group and the sutures group respectively. Several studies reported similar success rates. In the study published by J-P Lucot et al. the success rate was ranging from 61 to 81% [20], while Iliano et al. reported a 100% success rate of apical compartment correction in 50 patients after a 24-month follow-up period [21], Oudheusedn et al. showed a success rate that ranged from 89.3 to 90% [22] after a 12-month follow up period, and Cortes et al. showed a 96% anatomical success rate after a 3-year follow-up period [23]. The glue group showed a significantly (*p* = 0.02) better correction of anterior compartment points Aa and Ba – 3 vs. – 2 for the sutures group at 12 months, and at 24 months, a trend toward a better correction of points Aa and Ba was found in the glue group without reaching statistical significance. This could be explained by the low number of patients (27 patients per group) and probably a larger group of patients would have shown a statistically significant difference between both groups. This raises the question of whether a greater number of patients should be recruited in future randomized studies.

Functional results, as assessed by the quality of life score, were significantly improved in both groups at 1, 12, and 24 months post-operatively, indicating that the two fixation methods presented similar clinical outcomes. Quality of life scores were also significantly improved in other studies [20], Iliano et al. and Panel et al. also reported a significant improvement in PDFI-20 and PSDQ-7 at 3 and 2 months [15]. Interestingly, sexual function (assessed by PISQ-12) did not change post-operatively in either group, suggesting that the mesh fixation method may not influence sexual outcomes in this population of patient. However, the study conducted by Salomon et al. showed significant improvement in sexual function, as expressed by PISQ-12 scores in a prospective study that included 81 patients [24]. Similarly, Rusavy et al. reported significant improvement in sexual function and in PISQ-12 scores 1 year (36.0 vs. 38.1, *p* < 0.01) after laparoscopic surgery in a cohort of 333 women [25]. Our sexual function results could be due to the subjective reporting of the PISQ-12 scores and due to the small cohort (27 patients per group).

The incidence of urinary incontinence was also similar between both groups without any significant difference at 1 or 24 months. At 24 months, no case of de novo stress urinary incontinence was reported herein. Other studies have reported an incidence < 1% of de novo stress urinary incontinence. De novo stress incontinence was reported in 0.7% cases following laparoscopic sacrocolpopexy in the study including 137 patients conducted by Christmann-Schmid et al. [26]. Similarly, Iliano et al. reported no case of de novo stress incontinence after a 24-month follow-up period in 63 patients who underwent laparoscopic sacrocolpopexy [27]. No case of mesh erosion or exposure was reported, which are common concerns in mesh-related procedures. This highlights the safety of both surgical techniques, since glue mesh fixation proved to be effective and safe regarding long-term complications. Other studies reported different rates of mesh exposure: Dabica et al. reported a 0–6% mesh exposure rate after laparoscopic sacrocolpopexy after a 12-month follow-up period [28] and Izett-Kay et al., did not find mesh exposure after a 7-year follow-up period [29]. Several other studies reported no mesh exposure after a 1-year follow-up period using polypropylene mesh [30]. Salomon et al. reported in their study that the use of lightweight polypropylene mesh might have an effect in decreasing mesh exposure rate [31]. The mesh used herein is a polypropylene mesh, which could explain our 0% mesh exposure and erosion rate.

The strength of this study is the single center prospective randomized controlled trial design, including patients anonymously randomized to two groups and with an in-depth reporting of results. A relatively significant follow-up period, extending to 24 months post-operatively, and without having any patient lost-to-follow-up are considered important points of strength. Only one experienced surgeon who has already overcome his learning curve (more than 50 procedures), operated on all the patients, in one single center, with the same peri-operative conditions, contributing to decreasing the difference that might affect the results. All this adds to the homogeneity of the study and contributes to its strength.

However, our study also has several limitations. The small size of the cohort (only 27 patients per group) may limit the interpretability of the results or potentially skew them in a specific direction. Additionally, the single-center design may have limited the recruitment process, resulting in a lower diversity in the cohort, and limiting the generalizability of the findings to a broader population. The use of an anterior and/or a posterior mesh instead of a standardized Y-mesh for all patients could also have been a source of confounding factor and one of the limiting factors.

## Conclusion

In conclusion, the use of synthetic glue Ifabond™ in laparoscopic sacrocolopexy is a safe alternative to suturing. It allows, as the suturing technique, a good anatomical correction of POP and an improvement of the quality of life. While it did not result in a shorter overall operative time in the hands of an experienced surgeon, it did significantly reduce the time required for anterior mesh fixation and led to equivalent outcomes, which is of considerable interest to surgeons who are interested in implementing the laparoscopic sacrocolpopexy technique.

Given the similar recurrence and complication rates, safety profile, and functional results, the use of synthetic glue for mesh fixation is considered a safe and promising alternative especially for young surgeons, although more studies with larger cohorts and a longer follow-up period, as well as studies including a Y-mesh instead of an anterior and/ or a posterior mesh need to be conducted to assess long-term efficacy and complication rate.

## Supplementary Information

Below is the link to the electronic supplementary material.

Supplementary Material 1

## Data Availability

The data that support the findings of this study are not openly available but are available from the corresponding author upon reasonable request.
